# Dynamic Ionic Environment Modulation for Precise Electrosynthesis of Heterostructured Bimetallic Nanoparticles

**DOI:** 10.1002/advs.202415727

**Published:** 2025-03-10

**Authors:** Heekwon Lee, Xun Zhan, Jamie H. Warner, Hang Ren

**Affiliations:** ^1^ Department of Chemistry The University of Texas at Austin Austin Texas 78712 USA; ^2^ Texas Materials Institute The University of Texas at Austin Austin Texas 78712 USA; ^3^ Walker Department of Mechanical Engineering The University of Texas at Austin Austin Texas 78712 USA; ^4^ Allen J. Bard Center for Electrochemistry The University of Texas at Austin Austin Texas 78712 USA

**Keywords:** bimetal, electrodeposition, heterostructure, nanoparticles, scanning electrochemical cell microscopy

## Abstract

Bimetallic heterostructures, including core–shell and Janus configurations, often offer unique electrocatalytic properties compared to monometallic nanoparticles. However, achieving precise control over both elemental composition and spatial arrangement within these structures remains a challenge. Here, an electrosynthesis method is introduced that enables the fabrication of heterostructured bimetallic nanoparticles with precise, independent control of their elemental distribution. By leveraging dual‐channel scanning electrochemical cell microscopy (SECCM), the local ionic environment is dynamically modulated in situ, adjusting the deposition bias between channels to achieve selective electrodeposition. This approach allows temporal control over the solution conditions within the SECCM droplet, facilitating the synthesis of multi‐layer core–shell nanoparticles with tunable thickness, number, and sequence of layers. This technique is demonstrated with Pt–Cu and Pt–Ni systems, synthesizing arrays of Cu@Pt and Pt@Cu core–shell structures, which are then screened for catalytic activity in hydrogen evolution (HER) and oxygen reduction (ORR) reactions. The high spatial resolution and on‐demand control over the composition and structure make this method well‐suitable for creating arrays of complex, multi‐metallic heterostructures, which is expected to accelerate the discovery of advanced electrocatalytic materials, offering a platform for efficient and scalable electrocatalyst screening.

## Graphical abstract

An electrosynthesis method is introduced for fabricating heterostructured bimetallic nanoparticles, providing precise and independent control over elemental distribution within individual nanostructures. By dynamically modulating the ionic environment in a dual‐channel nanopipette using scanning electrochemical cell microscopy, core–shell nanoparticles with customizable elemental hierarchy and thickness are achieved. The synthesized nanoparticles are readily screened for their electrocatalytic activities.

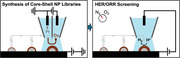

## 1 Introduction

Here, we report an electrosynthesis method for creating heterostructured bimetallic nanoparticles with precise, independent control over elemental distribution within each nanostructure. By dynamically altering the ionic environment in a dual‐channel nanopipette in SECCM, core–shell nanoparticles with independent control over deposition order and thickness are achieved. This method offers a platform for high‐precision synthesis of complex nanostructure libraries for advancing accelerated electrocatalyst discovery.

Metallic heterostructures—such as core–shell and multi‐layer configurations—have gained significant interest due to their unique optical, catalytic, and magnetic properties.^[^ ^1^, ^2^, ^3^, ^4^ ^]^ Bimetallic nanoparticles with heterostructures often exhibit enhanced catalytic activity for reactions like hydrogen evolution (HER), oxygen reduction (ORR), and methanol oxidation (MOR) compared to their monometallic counterparts.^[^ ^5^, ^6^, ^7^, ^8^, ^9^ ^]^ However, their activity is highly sensitive to both composition and atomic arrangement, even with identical atomic ratios, variations in atomic configuration can lead to markedly different properties in intermetallic compounds versus heterogeneous nanostructures.^[^ ^10^ ^]^

Although precise control over the size, shape, and composition of bimetallic heterostructures has advanced our understanding of structure‐function relationships, achieving continuous, sequential control over structural features remains challenging.^[^ ^5^, ^11^, ^12^, ^13^, ^14^ ^]^ This difficulty is particularly relevant when identifying optimal catalysts for specific reactions. Additionally, complex designs like multi‐shell or layer‐by‐layer structures typically require multi‐step synthesis methods, which often suffer from low throughput. For instance, a standard method for synthesizing bimetallic core–shell nanoparticles involves an initial synthesis of one metal, followed by the reduction of a second metal.^[^ ^3^, ^15^, ^16^, ^17^, ^18^ ^]^ Other techniques, such as galvanic replacement,^[^ ^6^, ^19^ ^]^ simultaneous reduction,^[^ ^20^ ^]^ and thermal annealing, have also been explored.^[^ ^21^, ^22^ ^]^ Despite advancements in synthesis techniques, achieving precise control over layer arrangement and component interaction within a single nanoparticle remains a challenge, particularly critical in designing catalysts for tandem electrocatalytic reactions. In addition, the current method often requires multiple processes per nanostructure, and the low throughput significantly hinders accelerated materials discovery. Furthermore, the hierarchy of the core–shell and the material selection is usually constrained by the standard reduction potential difference of the dissimilar metals in galvanic replacement or the equilibrium phase segregation due to intrinsic thermodynamic properties.^[^ ^23^ ^]^

Expanding beyond the challenges of conventional methods for synthesizing metallic nanostructures, electrochemical synthesis offers a versatile approach for producing metallic nanoparticles with compositions ranging from unary metals to high‐entropy alloys, allowing precise control over oxidation and reduction without external oxidizing or reducing agents.^[^ ^24^, ^25^ ^]^ By adjusting parameters such as potential and deposition time, it is possible to control the size, shape, and composition of metallic nanoparticles, ensuring well‐defined interfaces between synthesized materials and electrode surfaces.^[^ ^26^, ^27^ ^]^ Nonetheless, achieving independent control over features such as core size, shell thickness, shell number, and sequence remains challenging in complex nanostructures. Traditional electrochemical methods, including bulk solution electrosynthesis, limit sample throughput by restricting each experimental condition to a single sample preparation. Additionally, bulk electrodeposition faces limitations in systematically confining nucleation sites due to the random nature of nucleation events, underscoring the importance of spatially resolved nanoparticle arrays for studying localized electrocatalysis.

Scanning electrochemical cell microscopy (SECCM) has emerged as a promising technique for the electrodeposition of metallic nanoparticles with precise control over size and composition with high spatial resolution.^[^ ^28^, ^29^, ^30^ ^]^ We recently reported a dual‐channel SECCM‐based electrosynthetic method that precisely controls the elemental composition of bimetallic nanoparticles via nanofluidic manipulation between the two channels of the nanopipette.^[^ ^31^, ^32^ ^]^ In this study, we expanded this method to control the structural hierarchy of heterostructured nanoparticles, particularly core–shell configurations, by dynamically modulating the local environment within the SECCM droplet cell. This dual‐channel SECCM setup enables independent control over deposition order and thickness by adjusting the ionic environment—a capability not achievable with conventional methods. Such temporal modulation during deposition allows precise determination of the identity and thickness of each component in core–shell nanoparticles, which can be further expanded into multi‐layer core–shell structures. Arrays of Cu@Pt and Pt@Cu heterostructured nanoparticles with controlled shell thickness were synthesized and screened for electrocatalytic activity in HER and ORR. This methodology demonstrates the potential for high‐precision, serial synthesis of heterostructured nanoparticles, providing a platform for investigating interactions in tandem electrocatalysis.

## 2 Results and Discussion

Dual‐channel SECCM was used to dynamically control the solution environment for the deposition of bimetallic heterostructures. A dual‐channel glass nanopipette (aperture radius ≈360 nm, Figure S1, Supporting Information), filled with two different precursor solutions (e.g., Cu^2+^ and PtCl_4_^2−^), served as a probe. The pipette was positioned to approach the substrate, e.g., glassy carbon, while a substrate potential (*V*_sub_) was applied between the substrate and the quasi‐reference counter electrode (QRCE) within one of the channels. When the meniscus at the pipette apex made contact with the substrate, the circuit was completed, generating a sudden current flow. This current provided feedback to stabilize the pipette in place while maintaining droplet contact, enabling electrodeposition at a precisely defined local electroactive area. The potential applied to the substrate (vs one QRCE), *V*_sub_, controlled the driving force for electrodeposition, while the voltage bias between QRCEs in the channels (*V*_bias_) regulated precursor movement between channels via the droplet. The working electrode potential (*V*_WE_), which is the potential drop across the substrate/electrolyte interface, is approximated as *V*_sub_−0.5*V*_bias_ as reported in earlier studies.^[^ ^31^, ^32^ ^]^ As shown in our previous work, *V*_bias_ modulates precursor flux near the electrode surface through migration and electroosmotic flow, while the primary mass transport mode is migration when the electrolyte is acidic, which is the condition used in this work.^[^ ^32^ ^]^ At sufficient positive or negative *V*_bias_, one metal precursor can be effectively excluded from the droplet, allowing the deposition of nanoparticles with a single metal composition.^[^ ^31^ ^]^

**Scheme 1:**
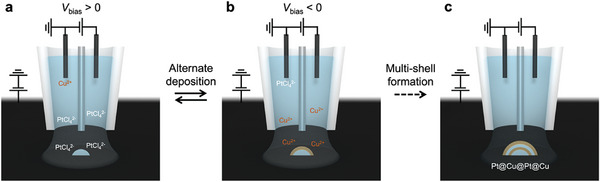
Schematic illustration of electrodeposition of heterostructured nanoparticles by dynamically changing the solution environment in the droplet of SECCM. a) Enrichment of PtCl_4_^2‐^ within a droplet under a positive potential bias. b) Enrichment of Cu^2+^ under a negative potential bias. c) Formation of a Pt@Cu@Pt@Cu nanoparticle by alternate deposition.

Dynamic control of the precursor concentrations near the electrode in SECCM during deposition enables the synthesis of complex hierarchical nanostructures. The concept is depicted in **Scheme** **1**, using the deposition of Pt‐core Cu‐shell nanoparticle (Pt@Cu) as an example. The left channel of the dual‐channel nanopipette is filled with a solution containing Cu and Pt precursors (1 mm Cu^2+^ and 1 mm PtCl_4_^2−^ in 2 mm HClO_4_), while the right channel contains only 2 mm HClO_4_. Both Cu and Pt precursors are placed in the same channel because they migrate in opposite directions under an applied potential bias (*V*_bias_) between the QRCEs (right side vs left side). Initially, a positive *V*_bias_ is applied, causing Pt precursor (PtCl_4_^2−^) to migrate from the left to the right channel, while the Cu precursor (Cu^2+^) recedes into the bulk of the left channel, as shown in Scheme 1a. Electrodeposition at the substrate then generates Pt nanoparticles serving as the cores. The local ionic environment is then dynamically adjusted by applying a negative *V*_bias_, which enriches Cu^2+^ in the droplet, allowing a Cu layer to be deposited on the Pt core, forming Pt@Cu core–shell nanoparticles (Scheme 1b). Similarly, Cu‐core Pt‐shell (Cu@Pt) nanoparticles can be fabricated by reversing the *V*_bias_ sequence during deposition. Additionally, multi‐layer core–shell nanoparticles can be synthesized by oscillating *V*_bias_ to dynamically modulate the solution environment during deposition, as illustrated in Scheme 1c. All SECCM experiments were performed in a custom‐built environmental chamber to control the atmosphere near the droplet to be either humidified N_2_ or O_2_ gases depending on the reaction of interest (see Section S2, Supporting Information).^[^ ^33^ ^]^

We experimentally validated the electrodeposition of heterostructures in the Cu‐Pt system. Cyclic voltammograms under SECCM on glassy carbon substrate for a solution containing 1 mm Cu(ClO_4_)_2_, 1 mm K_2_PtCl_4_, and 2 mm HClO_4_ are shown in Figure S3 (Supporting Information). In the negative‐going scan at *V*_bias_ = −0.4 V (Figure S3a, Supporting Information), two prominent cathodic waves appear when *V*_WE_ is lower than −0.5 V (vs Pt pseudo‐reference electrode), corresponding to the reduction of Cu and proton, respectively. In the positive‐going scan, anodic stripping of Cu is observed when *V*_WE_ is above −0.5 V. As shown in Figure S3b,c (Supporting Information) the charge associated with Cu stripping decreases as *V*_bias_ increases and becomes negligible at *V*_bias_ = 0.4 V. At this high positive bias, the resulting nanoparticles are mostly composed of Pt. Additionally, the cathodic current waves associated with the Pt reduction are dominant at *V*_bias_ = 0.4 V, and Cu deposition becomes absent (Figure S3c, Supporting Information). The assignment of the voltammetric waves was further confirmed by macroelectrode voltammetry on glassy carbon (3 mm dia.) and a Pt disk electrode (2 mm dia.) in different precursor solutions (Figures S4 and S5, Supporting Information). Overall, this dynamic modulation of *V*_bias_ in SECCM allows precise control over the Cu‐Pt composition, enabling independent deposition of each element.

**Figure 1:**
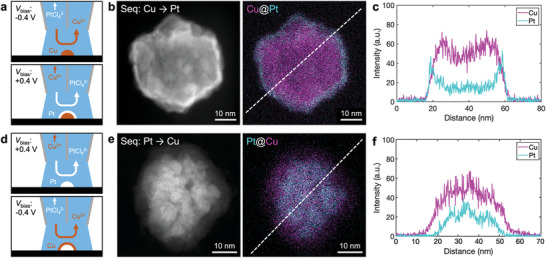
Dynamic modulation of solution for electrodeposition of Cu–Pt core–shell nanoparticles in SECCM. a) Schematic of Cu@Pt electrodeposition. b) ADF‐STEM image and elemental map of a Cu@Pt nanoparticle, with c) the corresponding elemental intensity profile. d) Schematic of Pt@Cu electrodeposition e) ADF‐STEM image and an elemental map with f) the corresponding elemental line profile. Cu (Pt) deposition step: *V*_WE_ at −1.03 V (−1.15 V), *V*_bias_ at −0.4 V (+0.4 V). Deposition time for each step: 0.2 s were used for each step. *V*_bias_ is right versus left channel.

Deposition of a Cu@Pt core–shell nanoparticle was achieved by first depositing at *V*_bias_ of −0.4 to form a Cu‐enriched (≈99% mole fraction) nanoparticles core, immediately followed by deposition at *V*_bias_ of 0.4 V, to form the Pt shell, as illustrated in **Figure** **1**a. The exact experimental sequence, including the *V*_sub_ and *V*_bias_ versus time, for the electrodeposition of Pt@Cu nanoparticles is shown in Scheme S1 (Supporting Information). Annular dark field‐scanning transmission electron microscopy (ADF‐STEM) and energy‐dispersive X‐ray spectroscopy (EDS) were used to verify the elemental distribution within the heterostructured bimetallic nanoparticles. As shown in Figure 1b,c, the electrodeposited particle exhibits a Pt shell surrounding a Cu core as expected. Alternatively, the core and shell of the nanoparticle were reversed to produce a Pt@Cu core–shell nanoparticle by simply reversing the deposition order, while keeping other conditions the same as illustrated in Figure 1d. The EDS images of Cu@Pt core–shell nanoparticles are shown in Figure 1e,f, confirming the expected Cu exterior layer with a Pt core.

**Figure 2:**
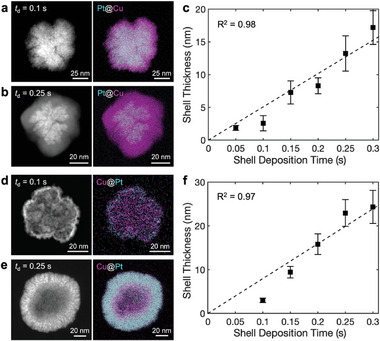
Control of shell thickness in Cu‐Pt core–shell nanoparticles. a,b) STEM images and EDS maps of Pt@Cu core–shell nanoparticles with shell deposition times (*t*_d_) of 0.1 s and 0.25 s. c) Average Cu shell thickness as a function of deposition time (*t*_d_). The dashed line is the linear fit. Error bars represent the standard deviation from n = 4. d,e) STEM and EDS maps of Cu@Pt core–shell nanoparticles with *t*_d_ of 0.1 s and 0.25 s. f) Average Pt shell thickness as a function of *t*_d_. The dashed line is the linear fit. Error bars represent the standard deviation from n = 4. *V*_WE_ = −1.03 (−1.15 V) and *V*_bias_ of −0.4 V (+0.4 V) were used for Cu (Pt).

The shell thickness of the nanoparticles can be precisely controlled in dual‐channel SECCM by controlling the charge, here by varying the deposition time, as demonstrated for Pt@Cu core–shell nanoparticles. Pt cores were first fabricated with a deposition time of 0.2 s, followed by the deposition of Cu shells with varying times. TEM‐EDS analysis in **Figure** **2**a,b shows as the deposition time increases from 0.05 to 0.25 s, the thickness of the Cu shell increases linearly from 1.9 to 17 nm, as shown in Figure 2c. This linear trend corresponds to a shell deposition rate of 51 nm s^−1^. Similar control over Pt shell thickness is achieved for Cu@Pt nanoparticles. As shown in Figure 2d–f, the Pt shell thickness also increases from 3 to 24 nm on average when shell deposition time varies from 0.1 to 0.3 s. These results demonstrate that precise control of shell thickness is feasible for different sequences of bimetallic shells. Note that deposition rates vary due to differing reaction rates under potentiostatic conditions and the competing HER. The slopes obtained here provide general growth rates, as no specific growth model is assumed.

**Figure 3:**
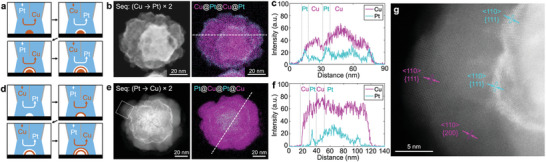
Electrodeposition of multi‐layer Cu–Pt nanoparticles. a) Schematic of electrodeposition process for a Cu@Pt@Cu@Pt multi‐layer nanoparticle. b) STEM images and EDS maps. c) Corresponding elemental intensity line profile. d) Schematic of the electrodeposition process for a Pt@Cu@Pt@Cu multi‐layer nanoparticle. e) STEM images and EDS maps. f) Corresponding elemental intensity line profile. g) High‐resolution STEM image of (a) showing the lattice structures of the exterior Cu and Pt shells.

The precision control in SECCM electrodeposition is further experimentally demonstrated through the construction of multi‐layer nanostructures, such as Cu@Pt@Cu@Pt and Pt@Cu@Pt@Cu multi‐layer core–shell nanoparticles. As shown in **Figure** **3**a, a Cu‐enriched core was first deposited, followed by alternating electrodeposition of Pt, Cu, and Pt, each under the same deposition conditions used previously. Analysis of STEM‐EDS maps in Figure 3b–c confirmed the hierarchy of the Cu@Pt@Cu@Pt multi‐layer core–shell structure. Similarly, Pt@Cu@Pt@Cu multi‐layer nanoparticle was synthesized as illustrated in Figure 3d: a Pt‐enriched core was formed first, followed by alternating deposition of Cu, Pt, and Cu shells. The STEM‐EDS analysis in Figure 3e,f shows the expected elemental distribution for the Pt@Cu@Pt@Cu multi‐layer nanoparticle. Moreover, high‐resolution STEM imaging of the shells of the Pt@Cu@Pt@Cu nanoparticle reveals distinct crystal orientations from Pt and Cu, displaying {111} and {200} planes along the <110> zone axis (Figure 3g, white dashed box in Figure 3e). This layer‐by‐layer multi‐shell construction highlights the advanced capability of SECCM in achieving precise control over composition and architecture in heterostructured nanoparticles through dynamic modulation of solution.

**Figure 4:**
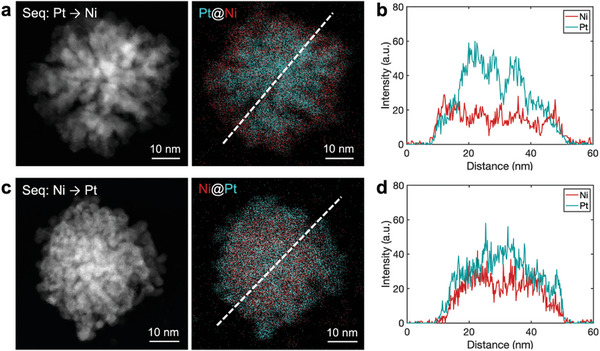
Electrosynthesis of Pt@Ni and Ni@Pt nanoparticles by SECCM. a) STEM and elemental images of a Pt@Ni nanoparticle. b) EDS intensity profile along the line in (a). c) STEM and elemental images of a Ni@Pt nanoparticle. d) EDS intensity profile along the line in (c). *V*_WE_ = −1.85 V (−1.15 V) and *V*_bias_ = −0.4 V (+0.4 V) were used for the deposition of Ni (Pt).

Electrodeposition of bimetallic heterostructures can be extended with other elemental combinations. We demonstrated the electrodeposition of Pt‐Ni heterostructures with two configurations: Pt@Ni and Ni@Pt. First, Pt‐enriched nanoparticles were created by reducing PtCl_4_^2−^ at *V*_WE_ = −1.15 V and *V*_bias_ = 0.4 V. The potentials were then changed to *V*_WE_ = −1.85 V and *V*_bias_ = −0.4 V to deposit a Ni layer on the Pt core, forming the Pt@Ni nanoparticles. The Pt@Ni nanoparticles were analyzed by EDS (**Figure** **4**a), with an elemental profile across the nanoparticle shown in Figure 4b, confirming the Ni shell around the Pt core. Conversely, Ni‐enriched cores were first deposited at *V*_WE_ = −1.85 V and *V*_bias_ = −0.4 V, followed by Pt deposition on the Ni core. The resulting Ni@Pt nanoparticles were analyzed by EDS as shown in Figure 4c,d, confirming the Pt shell around the Ni core. Additional examples are provided in Figure S6 (Supporting Information).

**Figure 5:**
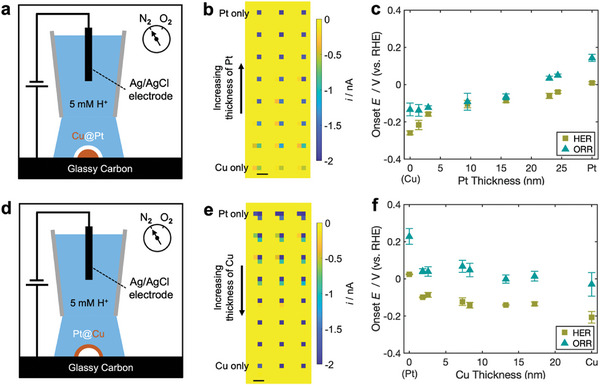
Electrocatalytic screening of HER and ORR activity on core–shell nanoparticles arrays with varying shell thickness. a) Schematic of single‐channel SECCM on a Cu@Pt nanoparticle. b) HER current heat map at −0.41 V versus RHE of Cu@Pt nanoparticle array with varying thickness across the row under N_2_. Scale bar: 5 µm. c) Onset potential at geometric current density (*j*_geo_) of −10 mA cm^−2^ versus Pt shell thickness for HER and ORR. d) Schematic of single‐channel SECCM on a Pt@Cu nanoparticle. e) HER current heat map at −0.41 V versus RHE of the Pt@Cu nanoparticle array with varying Cu shell thickness under N_2_ (scale bar: 5 µm). f) Onset potential versus Cu shell thickness for HER and ORR. A single‐channel pipette filled with 5 mm HClO_4_ is used in SECCM. ORR screening was performed with the same particle arrays in an environmental chamber saturated with O_2_ (current map not shown).

Finally, we demonstrate the screening of electrocatalytic activity of core–shell bimetallic nanoparticles using SECCM. HER and ORR were selected for this screening due to the relevance of Pt‐based bimetallic nanoparticles in electrolyzers and fuel cells.^[^ ^34^, ^35^ ^]^ Figure 5a–c show the electrocatalytic activity screening of Cu@Pt core–shell nanoparticles with varying Pt shell thicknesses. Eight distinct Cu@Pt structures with varying Pt thicknesses from 0 to 25 nm were deposited on a glassy carbon substrate, and the nanoparticles in each row were fabricated under identical conditions to ensure structural consistency for reproducibility assessment. A single‐barrel pipette with a diameter of ≈3 µm, filled with 5 mm HClO_4_ was used as a scanning probe in SECCM to evaluate the HER and ORR activities of these Cu@Pt nanoparticles in voltammetry (**Figure** **5**a). The screening was performed inside a N_2_ and O_2_ environmental chamber, allowing the same particle array to be scanned for HER and ORR. Figure 5b shows the current heat map at −0.41 V versus RHE for Cu@Pt nanoparticles with different Pt shell thicknesses, and the electrocatalytic activity was further evaluated by the onset potential at −10 mA cm^−2^ (Figure 5c), calculated using projected geometric area analysis shown in Figure S7 (Supporting Information). The onset potential for both HER and ORR shifts positively with Pt shell thickness. Raw voltammograms showing HER and ORR activity are provided in Figure S8 (Supporting Information). In Cu@Pt nanoparticles, a Pt shell of only a few nanometers (≈3 nm) significantly enhanced HER activity, while ORR activity remained unaffected by the Pt presence.

Similarly, Pt@Cu nanoparticles with varying Cu shell thickness were prepared for electrocatalytic screening (Figure 5d–f). The Cu shell thickness varied along each column in the array, with each row prepared under identical deposition conditions. Figure 5e shows the current heat map at −0.41 V versus RHE for HER on the Pt@Cu arrays. Similar trends in HER and ORR were observed for Pt@Cu nanoparticles, showing shifts from positive to negative as the Cu shell thickness increases, as seen in the local voltammograms in Figure S9 (Supporting Information). We further performed Tafel analysis on the local voltammograms using the negative‐sweeping scans (see Section S8, Supporting Information for the discussion about the analysis). Note that the SECCM voltammetry under our study reached a steady state, as indicated by the sigmoidal shape of the voltammogram, simplifying the Tafel analysis (Figure S10a, Supporting Information). The exchange current in HER for Cu@Pt and Pt@Cu nanoparticles exhibits a consistent trend with the onset potential: HER kinetics increase with Pt shell thickness in Cu@Pt, and dramatically decrease with the addition of Cu shell in Pt@Cu (Figure S11, Supporting Information). The Tafel slopes for both types of particles are relatively stable, ranging between ≈90 and ≈120 mV dec^−1^. When the particle is purely Cu, or the outer layer has thick Cu (i.e., Pt@Cu with > 10 nm shell), the Tafel slope is <100 mV dec^−1^. On the other hand, the Tafel slope increases to 120 mV dec^−1^ as the surface Pt component increases (Figure S10b,c, Supporting Information). Moreover, we performed bulk electrodeposition of Cu@Pt and Pt@Cu nanoparticles on a glassy carbon substrate, which exhibited a similar trend of increasing and decreasing onset potentials for HER and ORR depending on their elemental configurations (see Section S9, Supporting Information for bulk electrodeposition results). Overall, a few nanometers of shell thickness can significantly influence the electrocatalytic activities of HER and ORR.

## 3 Conclusion

In conclusion, we have developed a versatile electrosynthesis method for heterostructured bimetallic nanoparticles that enables precise, independent control over elemental distribution, and structural hierarchy. By leveraging dynamic modulation of the ionic environment within a dual‐channel SECCM setup, we achieved selective electrodeposition with control over deposition order, shell thickness, and layer sequence. This approach facilitates the fabrication of complex core–shell and multi‐layer structures, as demonstrated with Cu@Pt, Pt@Cu, Pt@Ni, and Ni@Pt configurations, and the thickness of the shell can be controlled with a precision of nanometers. In addition, multi‐layer nanoparticles, e.g., Cu@Pt@Cu@Pt and Pt@Cu@Pt@Cu, can be synthesized. This method represents a great advancement in throughput, facilitating both the rapid synthesis of nanoparticles and the screening of their catalytic activities (see Sections S10 and S11, Supporting Information for a detailed discussion). The Cu@Pt and Pt@Cu core–shell nanoparticles were prepared in arrays with varying shell thickness, allowing fast screening of electrocatalytic activity in HER and ORR within one SECCM experiment. Future studies could leverage this methodology to systematically investigate how layer order, thickness, and elemental distribution within heterostructures influence catalytic pathways in tandem reactions, thereby paving the way for tailored catalysts with enhanced selectivity and efficiency. While this method is demonstrated with the dual‐barrel configuration, it can be extended to a multi‐channel pipette with three or more barrels, enabling the exterior structures to be decorated with multiple elemental layers, creating a library of multi‐metallic heterostructures. The adaptability of the method to different elemental combinations, coupled with high spatial resolution, holds significant potential for targeted synthesis and screening of nanostructure libraries, which are critical for advancing the accelerated discovery of next‐generation electrocatalysts for fuel cells and electrolyzers. Moreover, once optimal structures are identified, traditional synthetic techniques can be employed for large‐scale production, offering a pathway to translate SECCM discoveries into practical and scalable catalyst manufacturing.

## 4 Experimental Section

### Chemical and Materials

Copper (Cu), platinum (Pt), and nickel (Ni) aqueous solutions were prepared with copper(II) perchlorate hexahydrate (Sigma–Aldrich), potassium tetrachloroplatinate(II) (BeanTown Chemical), nickel(II) chloride hexahydrate (Sigma–Aldrich), perchloric acid (70%, Alfa Aesar), and deionized water (18.2 MΩ cm, Synergy Water Purification System). Platinum wires (0.1 mm diameter, 99.9% metals basis, Alfa Aesar) were used as a quasi‐reference counter electrode (QRCE) in SECCM.

### Pipette Preparation

Dual‐channel nanopipettes were fabricated from theta quartz capillaries (QT120‐90‐7.5, Sutter Instrument) using the P‐2000 micropipette puller (Sutter Instrument). The pulling parameters of HEAT = 700, FIL = 4, VEL = 40, DEL = 130, PUL = 30 for line 1 and HEAT = 600, FIL = 3, VEL = 30, DEL = 130, PUL = 100 for line 2 were used.

### SECCM Measurements

The SECCM experiment was conducted on a custom‐built scanning electrochemical probe. The vertical movement of the pipette was controlled by a one‐axis piezo‐positioner (P‐622.1CD, Physik Instrumente), and the x‐y direction of the substrate was controlled by a two‐axis piezo positioner (P‐542.2CD. Physik Instrumente). The scanning probe system was mounted on a mechanical vibration isolator (Minus K Technology, BM‐4) and enclosed in a Faraday cage. Patch‐clamp amplifier (Dagan Chem‐clamp) was used to measure the current, and the data acquisition and instrument control was performed by using an FPGA card (PCIe‐7846R, National Instrument) interfaced with the Warwick Electrochemical‐Scanning Probe Microscopy Platform (WEC‐SPM) software, which was generously provided by the Warwick Electrochemistry & Interfaces Group (WEIG). For TEM analysis of synthesized nanoparticles, core/multi‐shell structures were directly deposited onto a carbon‐coated gold TEM grid (CF‐400‐Au, Electron Microscopy Sciences) to analyze their as‐prepared state. The gas atmosphere for synthesis and screening was controlled by a home‐built environmental chamber where humidified N_2_ or O_2_ could be introduced. The screening was done using a single‐channel pipette with a hopping distance of 2 µm.

### Transmission Electron Microscopy and Energy Dispersive Spectroscopy

Annular dark field‐scanning transmittance electron microscopy (ADF‐STEM) was performed using a JEOL NEOARM with a probe corrector for STEM operated at an accelerating voltage of 200 kV was used for nanoparticle imaging. EDS was conducted in the JEOL NEOARM equipped with a JEOL large‐angle silicon drift detector (0.96 sr). Map processing was performed with ThermoFisher Pathfinder software and analyzed using ImageJ.

## Conflict of Interest

The authors declare no conflict of interest.

## Supporting information



Supporting Information

## Data Availability

The data that support the findings of this study are available from the corresponding author upon reasonable request.
