# Supplementary material for: Dynamic Ionic Environment Modulation for Precise Electrosynthesis of Heterostructured Bimetallic Nanoparticles
Source: Adv Sci (Weinh). 2025 Mar 10;12(17):2415727. doi: 10.1002/advs.202415727 (PMC12061254; doi:10.1002/advs.202415727)
Supplement: Supplementary file 1 — Supporting Information [file ADVS-12-2415727-s001.pdf]

ADVANCED SCIENCE
Open Access

Supporting Information

for *Adv. Sci.*, DOI 10.1002/advs.202415727

Dynamic Ionic Environment Modulation for Precise Electrosynthesis of Heterostructured Bimetallic Nanoparticles

*Heekwon Lee, Xun Zhan, Jamie H. Warner and Hang Ren**

Supporting Information

Dynamic Ionic Environment Modulation for Precise Electrosynthesis of Heterostructured Bimetallic Nanoparticles

Heekwon Lee[1], Xun Zhan[2], Jamie H. Warner[2, 3], Hang Ren[1, 2, 4,*]

1. Department of Chemistry, The University of Texas at Austin, Austin, Texas 78712, United States

2. Texas Materials Institute, University of Texas at Austin, Austin, Texas 78712, United States

3. Walker Department of Mechanical Engineering, University of Texas at Austin, Austin, Texas 78712, United States

4. Allen J. Bard Center for Electrochemistry, University of Texas at Austin, Austin, Texas 78712, United States

[*]Corresponding author: hren@utexas.edu

Table of Contents

S1. Characterization of dual-channel nanopipettes........ ***S-2***
S2. Environmental chamber in SECCM........ ***S-3***
S3. Cyclic voltammetry of Cu, Pt, and Cu-Pt........ ***S-4***
S4. Sequential electrodeposition procedure in dual-channel SECCM........ ***S-7***
S5. Additional examples of Pt-Ni core-shell nanoparticles........ ***S-8***
S6. Geometric area analysis in local electrochemical mapping........ ***S-9***
S7. Linear sweep voltammograms in local electrochemical mapping........ ***S-10***
S8. Tafel analysis of heterostructured bimetallic nanoparticles........ ***S-12***
S9. Bulk electrodeposition of core-shell Cu-Pt nanoparticles........ ***S-13***
S10. Electrodeposition of a Cu-Pt nanoparticle array........ ***S-15***
S11. Quantitative comparison of throughput between SECCM and conventional techniques.. ***S-16***
S12. References........ ***S-17***

S1. Characterization of dual-channel nanopipettes

The opening size of the dual-barrel pipette was measured by scanning electron microscope (Scios2 Dual Beam SEM, Thermo Fisher Scientific) under 5 kV and 0.1 nA with a 7 mm working distance. The pipette has an opening of 360 nm for each aperture (**Figure S1**).

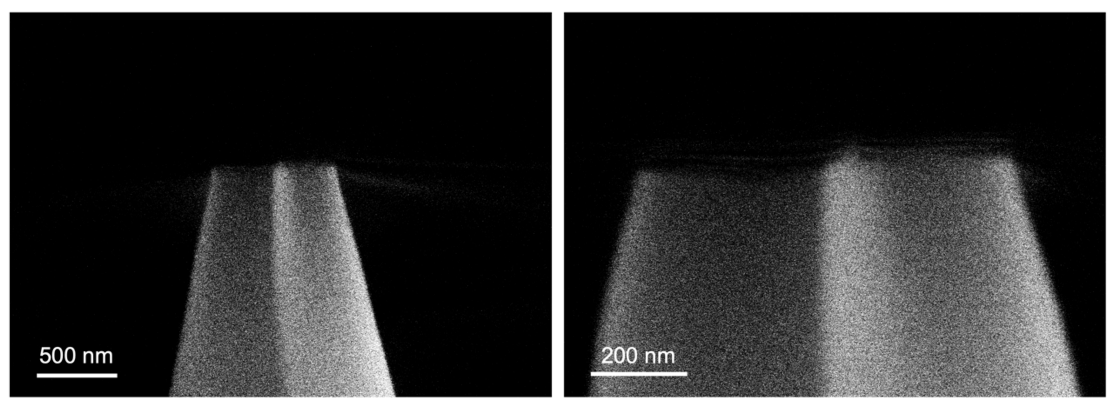

Figure S1. Scanning electron microscope (SEM) images of a dual-barrel pipette. The image on the right is a magnified view of the micrograph on the left.

S2. Environmental chamber in SECCM

Environmental control of electrodeposition of bimetallic nanoparticles was established using a gas flow system integrated with a chamber. N_2 or O_2 gas flowing at 80 cc/min was first passed through a DI water in a vial before being introduced into the chamber.

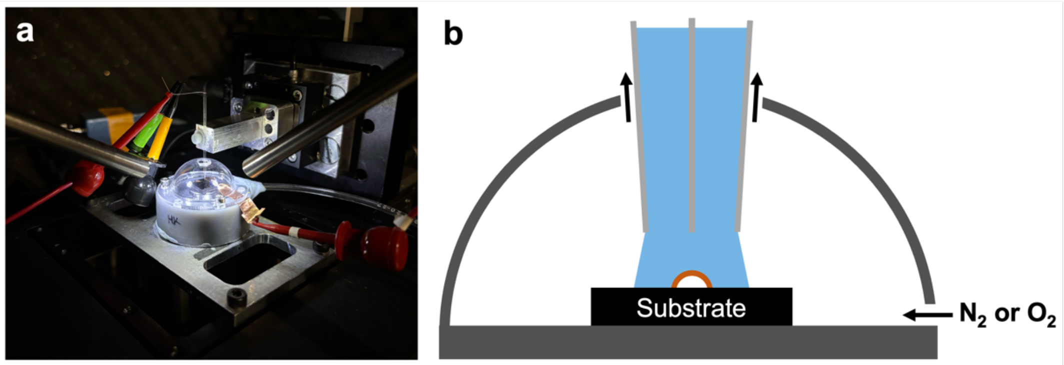

Figure S2. SECCM setup with an environmental chamber for bimetallic nanoparticle deposition and screening. (a) Photograph and (b) schematic illustration of the experimental configuration of SECCM, showing the pipette, substrate, and the environmental chamber with controlled N_2 or O_2 gas flow.

S3. Cyclic voltammetry of Cu, Pt, and Cu-Pt

For cyclic voltammetry in SECCM, both Cu and Pt precursor solutions (1 mM $Cu(ClO_4)_2$ + 1 mM K_2PtCl_4 + 2 mM $HClO_4$) were filled in the left lumen, while an acidic solution of 2 mM $HClO_4$ was filled in the right lumen. The substrate was glassy carbon, and the quasi-reference counter electrodes (QRCEs) were Pt electrodes. All voltammograms were collected with the same pipette, and V_{WE} was approximated as $V_{sub} - 0.5V_{bias}$ (**Figure S3**).

For bulk electrochemistry using a three-electrode system, 3 mm diameter glassy carbon disk electrodes served as the working electrode, with a silver/silver chloride electrode (Ag/AgCl) or a Pt wire as the reference electrode. The voltammograms for electrodeposition and oxidation of Cu, Pt, and their mixture solutions are presented in **Figure S4**. The shapes of the voltammograms are consistent regardless of the type of the reference electrode, and the potential shifts reflect the potential difference between the Ag/AgCl and the Pt quasi-reference electrodes.

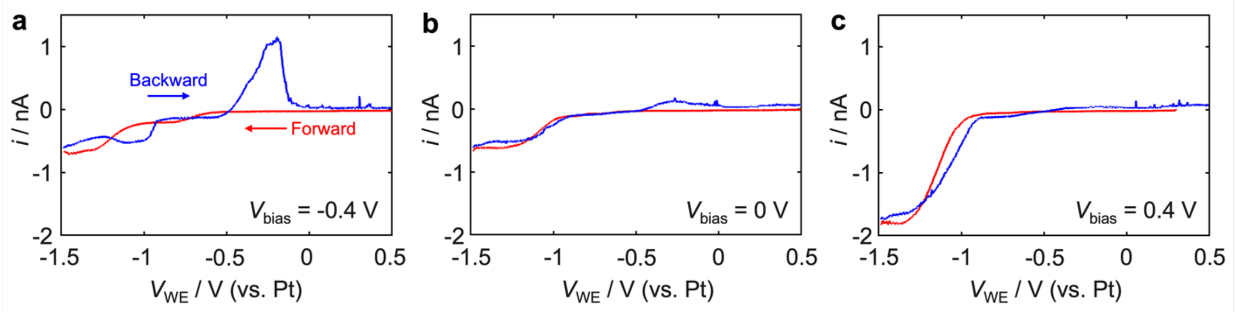

Figure S3. Cyclic voltammograms for the deposition and stripping of Cu–Pt particles in SECCM at different V_{bias} of (a) −0.4 V, (b) 0 V, and (c) 0.4 V. One barrel is filled with 1 mM $Cu(ClO_4)_2$, 1 mM K_2PtCl_4, and 2 mM $HClO_4$; the other barrel contains 2 mM $HClO_4$. Scan rate: 1 V/s.

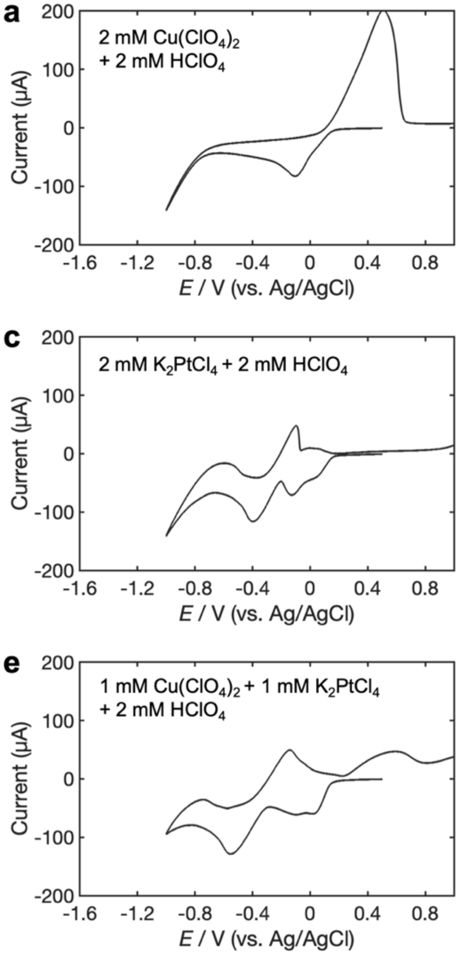

Figure S4. Macroelectrode voltammetry in solutions containing precursors of (a, b) Cu, (c, d) Pt, and (e, f) Cu-Pt with Ag/AgCl or Pt reference electrode. The solution compositions are described in each plot. Working electrode: glassy carbon (3 mm diameter), counter electrode: Pt, reference electrode: Ag/AgCl or Pt, scan rate: 0.1 V/s.

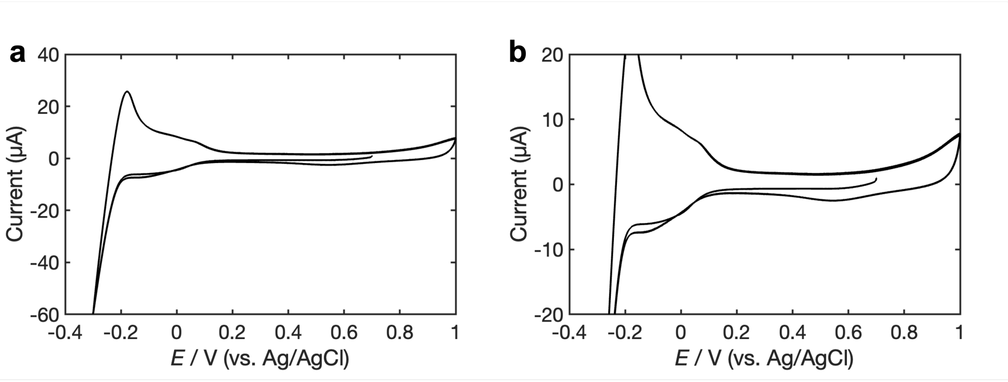

Figure S5. Macro-cyclic voltammetry of Pt in 5 mM HClO4. (a) Cyclic voltammogram showing the full range of current response and (b) magnified view of (a). Working electrode: Pt disk (2 mm

diameter), counter electrode: Pt coil, reference electrode: Ag/AgCl, scan rate: 0.1 V/s. Three consecutive cycles are shown with a starting E of 0.7 V.

S4. Sequential electrodeposition procedure in dual-channel SECCM

The electrodeposition of core-shell bimetallic nanoparticles was carried out in 5 steps. Firstly, the probe is positioned approximately 50 μm away from the substrate. During the approach (**Scheme S1a**), the substrate is held at V_{sub} = 0.3 V, and the potential between the barrel is at V_{bias}= 0 V. Once the droplet contacts the substrate, sequential electrodeposition is performed with precise nanofluidic control by V_{bias} and V_{sub} to achieve selective reduction of target elements. **Scheme S1b-c**, for example, illustrates the two-step fabrication process of Pt@Cu nanoparticles. Pt is first reduced to form the core (V_{bias} = 0.4 V and V_{sub} = -0.95 V), followed by Cu reduction to form the shell (V_{bias} = -0.4 V and V_{sub} = -1.15 V). This method can be expanded to have additional deposition sequences for multishell construction such as Pt@Cu@Pt@Cu nanoparticles, which is demonstrated in **Figure 3** in the main text. When deposition is completed, the probe is retracted (**Scheme S1d**) and then moves to the next position (**Scheme S1e**).

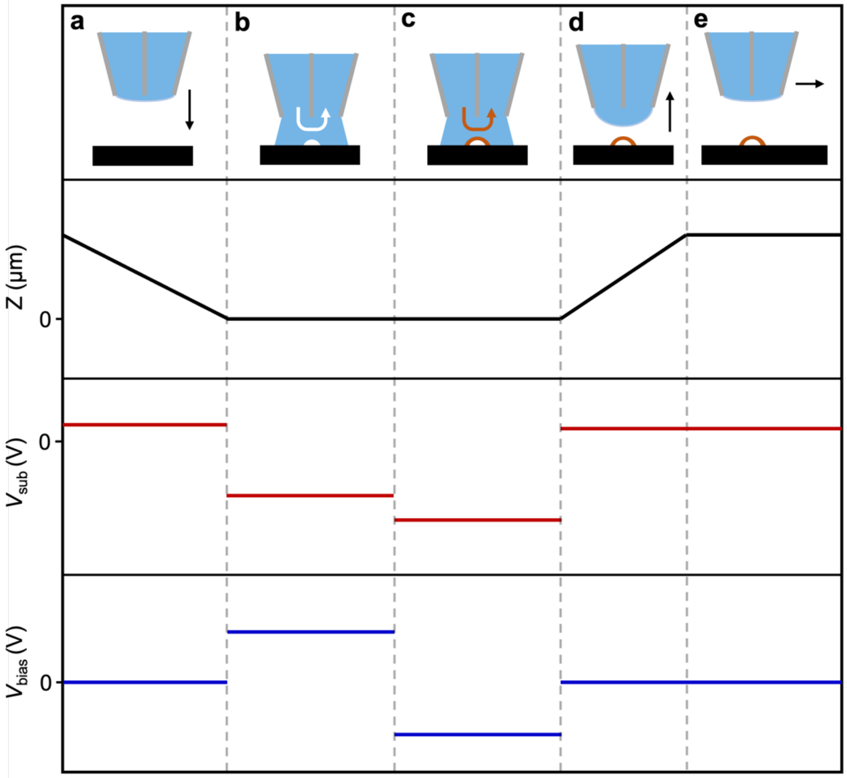

Scheme S1. Dual-barrel SECCM experimental sequence for electrodeposition of Pt@Cu nanoparticles. (a) approach and contact (b) Pt deposition (c) Cu deposition (d) retraction (e) X-Y move to the next position.

S5. Additional examples of Pt-Ni core-shell nanoparticles

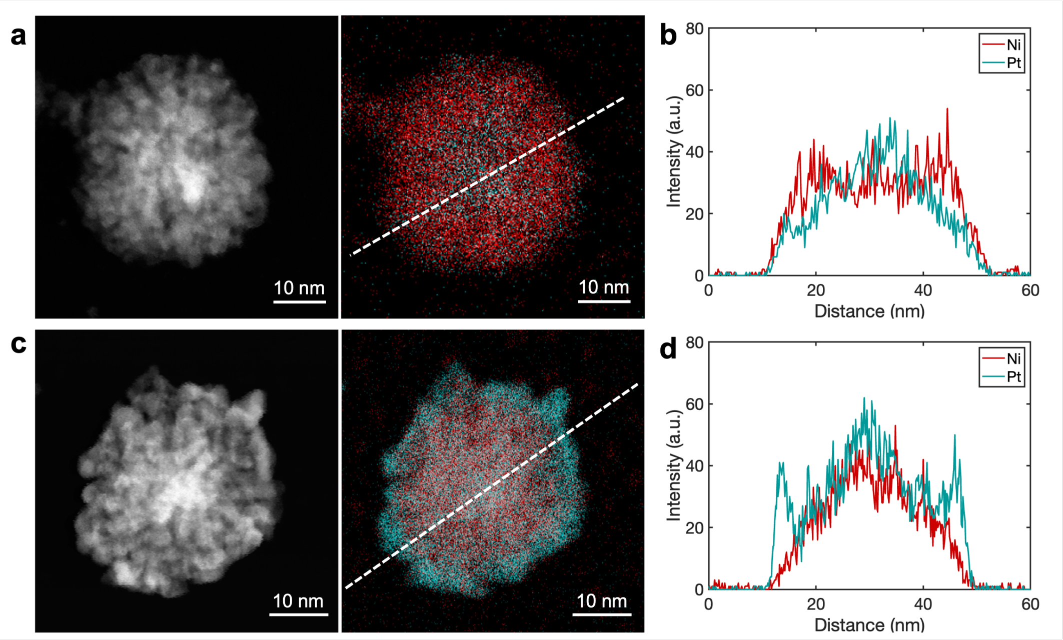

Figure S6. Elemental analysis of Pt-Ni core-shell nanoparticles synthesized by SECCM. (a) STEM and elemental images of a Pt@Ni nanoparticle. (b) EDS intensity profile along the line in (a). (c) STEM and elemental images of a Ni@Pt nanoparticle. (d) EDS intensity profile along the line in (c). V_{WE} = −1.85 V (−1.15 V) and V_{bias} = −0.4 V (+0.4 V) were used for the deposition of Ni (Pt).

S6. Geometric area analysis in local electrochemical mapping

The geometric area of nanoparticles in the nanoparticle array from SEM images is analyzed to normalize the catalytic current by MATLAB with *imbinarize* and *graythresh* functions. The approach starts with SEM image preprocessing, where the images are converted to grayscale and cropped to remove unwanted elements such as scale bars, ensuring a focus on the nanoparticles. After preprocessing, the nanoparticles are identified by applying an intensity threshold that distinguishes them from the background. Then, the area of each nanoparticle is quantified, converting the measurements to a physical scale based on the known pixel-to-length ratio as shown in **Figures S7b-c** and **S7d-e**. The total surface area of the nanoparticles in each image (or deposition spot) is calculated, providing the corresponding area for normalizing the electrochemical current. Note that this area represents the projected area of electrode active surface.

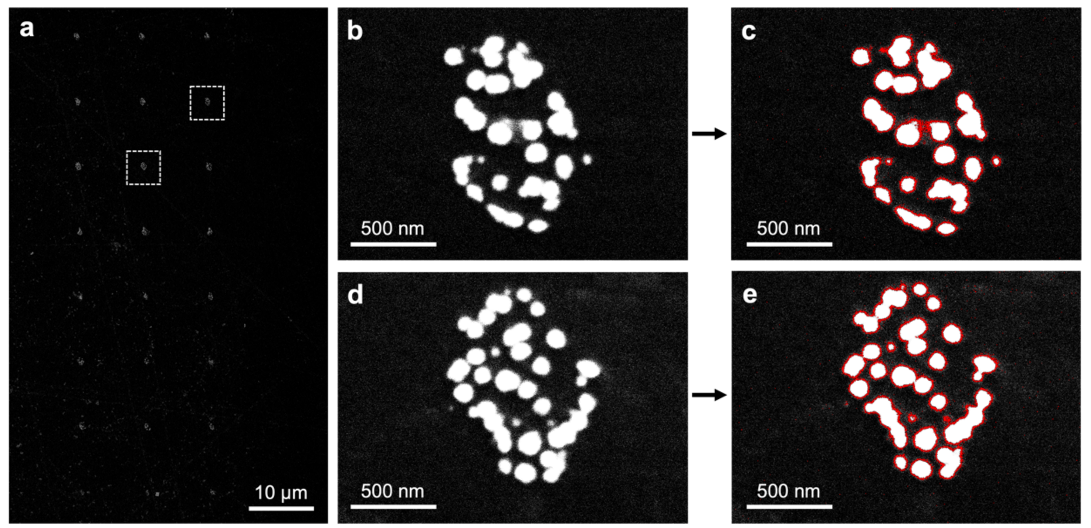

Figure S7. Geometric area analysis of a core-shell nanoparticle array. (a) SEM image of a nanoparticle array with varying shell thickness in Figure 5b. (b) SEM image of nanoparticles at a single spot, and (c) the corresponding detected surface area with red lines. (d) SEM image of another nanoparticle spot and (e) its measured surface area with red lines.

S7. Linear sweep voltammograms in local electrochemical mapping

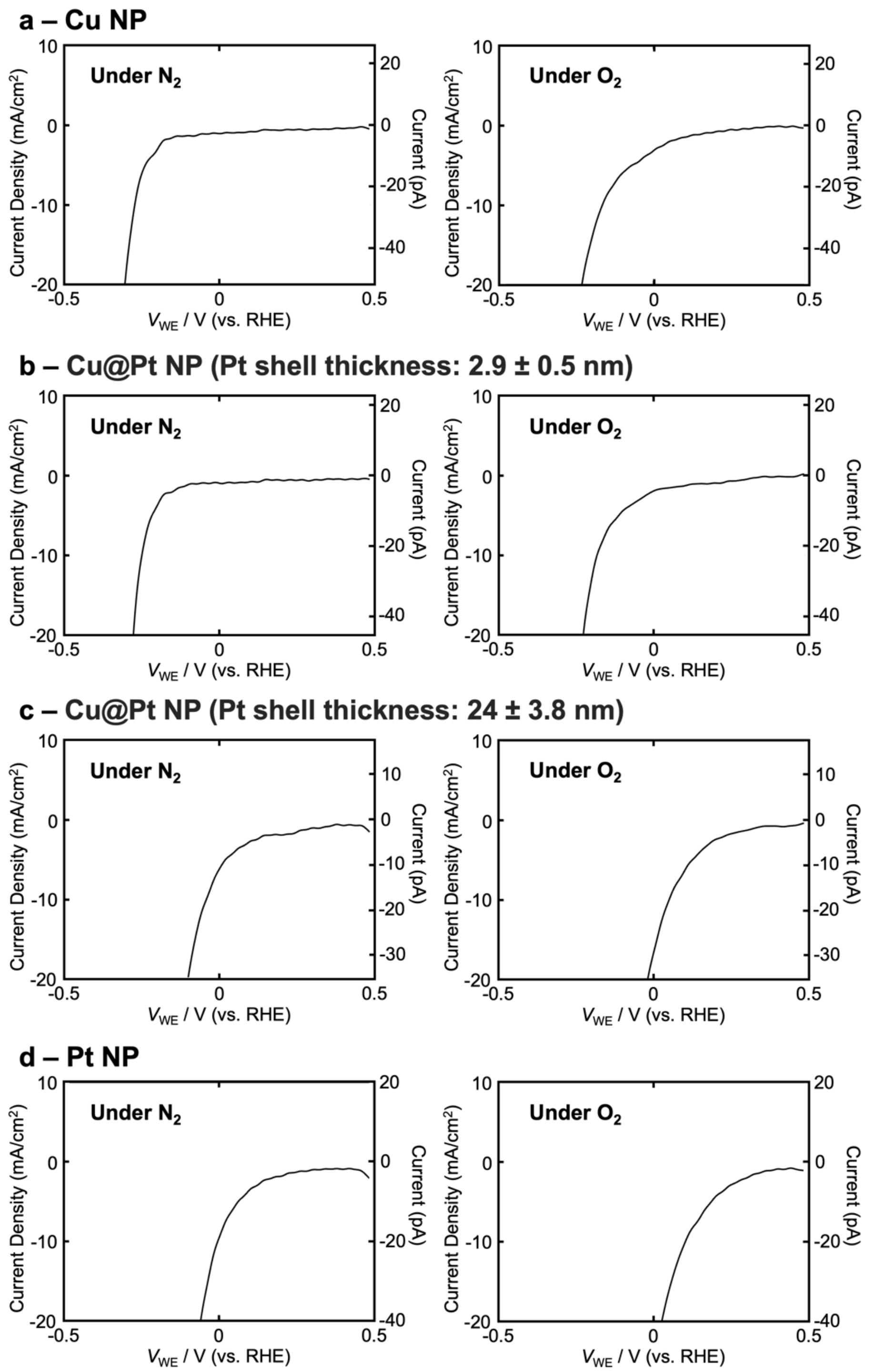

Figure S8. Linear sweep voltammograms of (a) Cu, Cu@Pt with various Pt shell deposition times: (b) 0.05 s and (c) 0.3 s. (d) Voltammogram of Pt nanoparticles. Voltammograms in the left column were conducted under the N_2 atmosphere to evaluate HER, and ones in the right column were performed under O_2 to evaluate ORR. Single-channel pipettes with 3-µm-diameter openings were used. The solution contains 5 mM $HClO_4$. The data is complementary to Figure 5b.

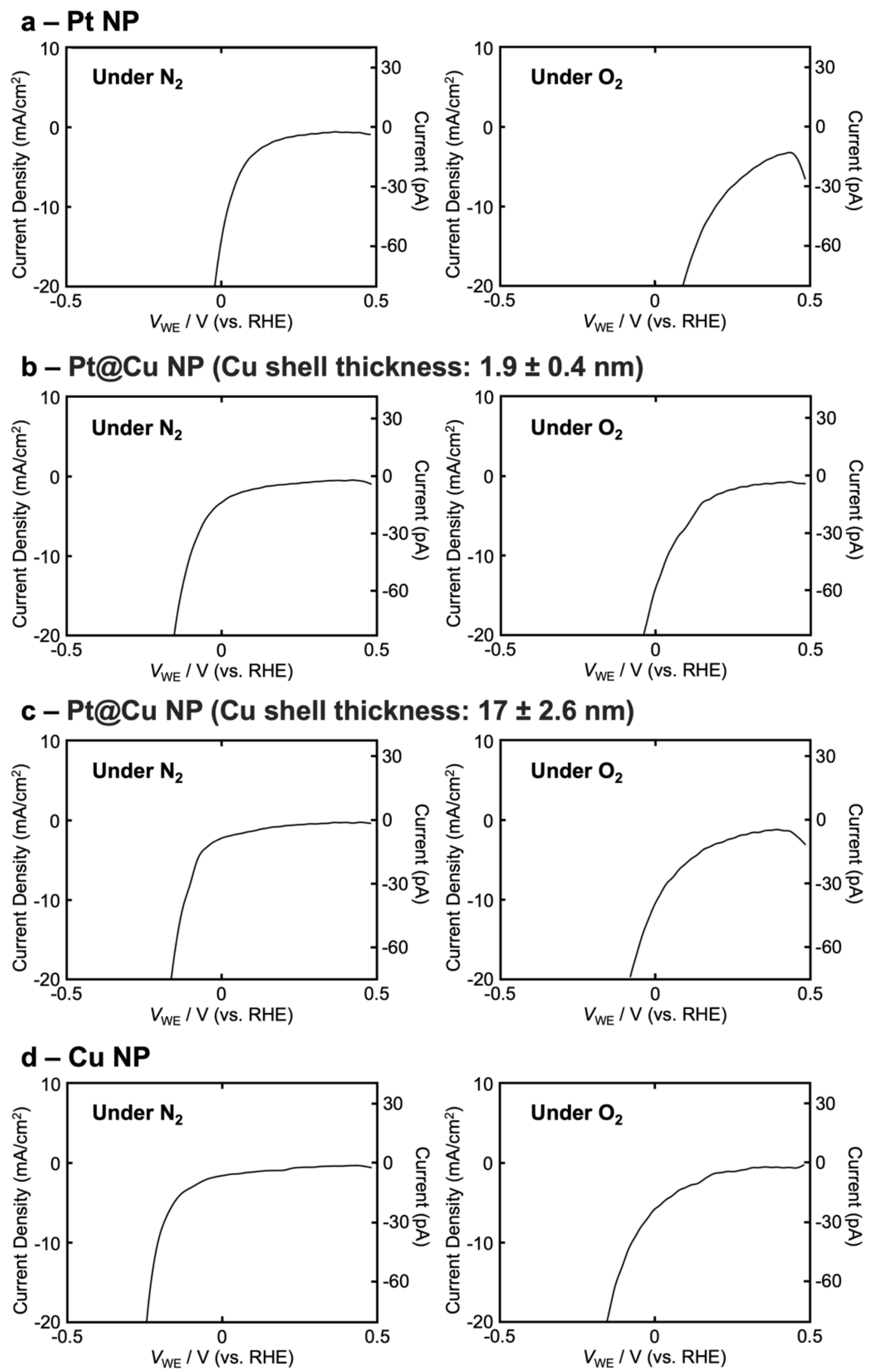

Figure S9. Linear sweep voltammograms of (a) Pt, Pt@Cu with various Cu shell deposition times: (b) 0.05 s and (c) 0.3 s. (d) Voltammogram of Cu nanoparticles. Voltammograms on the left column were conducted under the N_2 atmosphere to evaluate HER, and ones on the right column were performed under O_2 to evaluate ORR. Single-channel pipettes with 3-μm-diameter openings were used. The solution contains 5 mM $HClO_4$. The data is complementary to Figure 5e.

S8. Tafel analysis of heterostructured bimetallic nanoparticles

The Tafel slopes were analyzed using the kinetic currents on the local voltammograms from Figures 5b and e. The kinetic current was isolated from the total current based on the Koutecký–Levich equation.[1]

$$\frac{1}{i_{\mathrm{k}}} = \frac{1}{i_{\mathrm{total}}} - \frac{1}{i_{\mathrm{l}}} \qquad \text{(eq. 1)}$$

where i_{k} is the kinetic current, i_{total} is the total current, and i_{l} is the mass-transfer-limited current. Tafel slopes are extracted from i_{k}, which are shown in **Figure S10**.

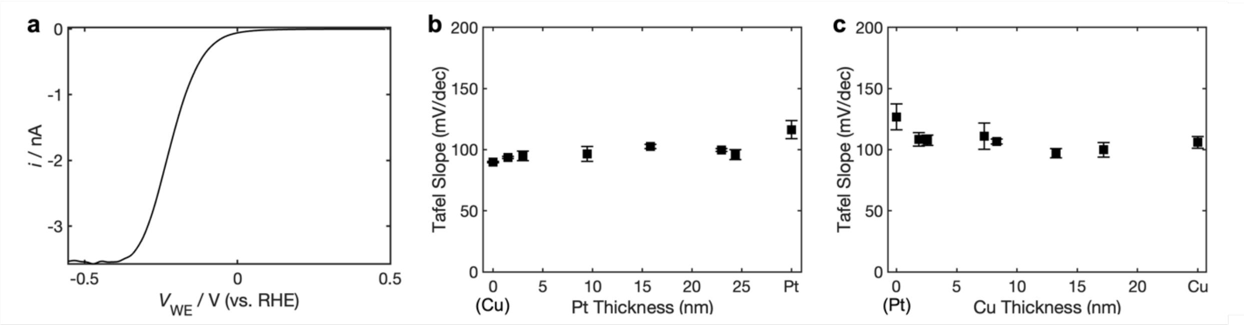

Figure S10. Tafel analysis of kinetic current in local electrochemical mapping of HER from Figure 5. (a) Exemplary LSV of Pt nanoparticles (b) Tafel slopes from Cu, Cu@Pt, and Pt nanoparticles from Figures 5a-c and (c) Pt, Pt@Cu, and Cu nanoparticles from Figures 5d-f.

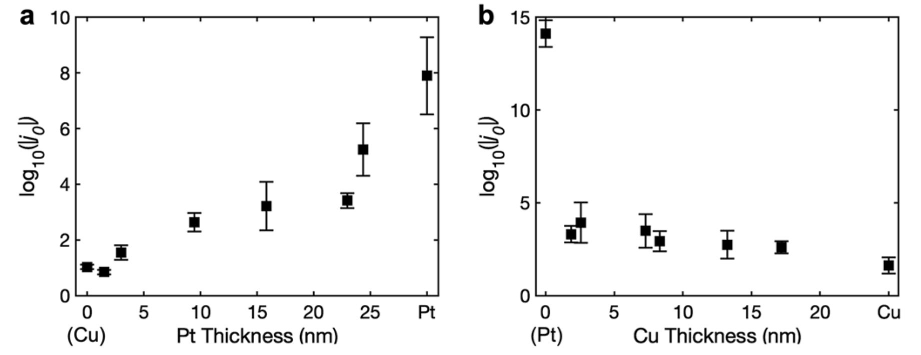

Figure S11. Comparison of exchange current density ($\log_{10}|j_0|$) (a) Cu@Pt nanoparticles from Figures 5a-c and (b) Pt@Cu nanoparticles from Figures 5d-f.

S9. Bulk electrodeposition of core-shell Cu-Pt nanoparticles

Firstly, core nanoparticles (Cu or Pt) were electrodeposited on glassy carbon substrate with an exposed area of 2 mm diameter, followed by a secondary deposition to grow the shell in a different solution with varying the deposition time to control shell thickness. Between deposition steps, we performed linear sweep voltammetry (LSV) to evaluate the catalytic performance for the hydrogen evolution reaction (HER) and oxygen reduction reaction (ORR) using 5 mM $HClO_4$ as the electrolyte. For Cu and Cu@Pt nanoparticles, as plotted in **Figure S12a**, the onset potential for both HER and ORR shifts positively with increasing Pt shell thickness (e.g., ORR at 0.7 V and HER at -0.3 V). Conversely, for Pt and Pt@Cu nanoparticles, the onset potential shifts from positive to negative as the Cu shell thickness increases (**Figure S12b**). Additionally, SEM images of Cu@Pt and Pt@Cu nanoparticles are presented in **Figure S13**. Deposition time of 10 sec was used for all steps for core and shells with $E_{\text{Pt-core}}$ of -0.4 V, $E_{\text{Cu-core}}$ of -0.4 V, $E_{\text{Pt-shell}}$ of 0 V, $E_{\text{Cu-shell}}$ of 0 V. All potential were referenced against a commercial Ag/AgCl electrode (3.4 M KCl).

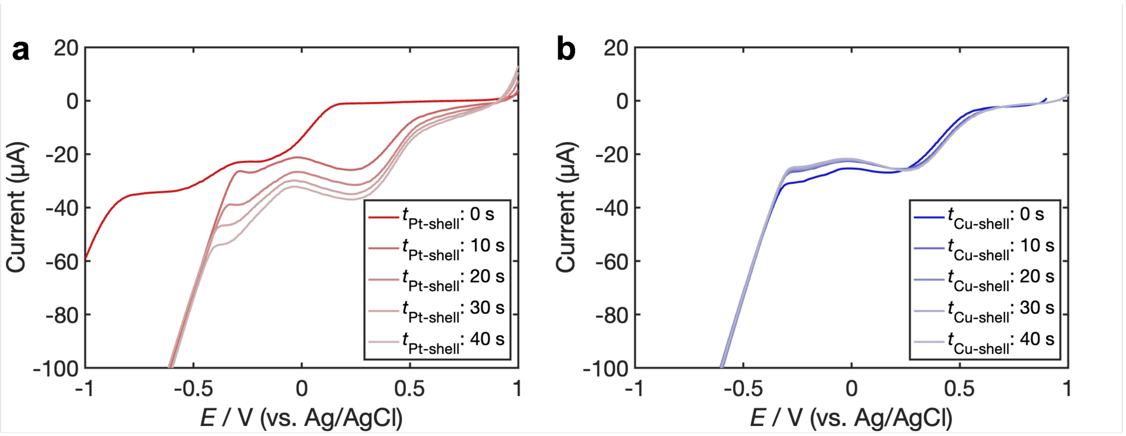

Figure S12. Linear sweep voltammetry for hydrogen evolution reaction (HER) and oxygen reduction reaction (ORR) on Cu–Pt nanoparticles: (a) Cu core with varying Pt shell thickness achieved by various deposition time. (b) Pt core with varying Cu shell thickness achieved by various deposition time. Scan rate: 0.1 V/s.

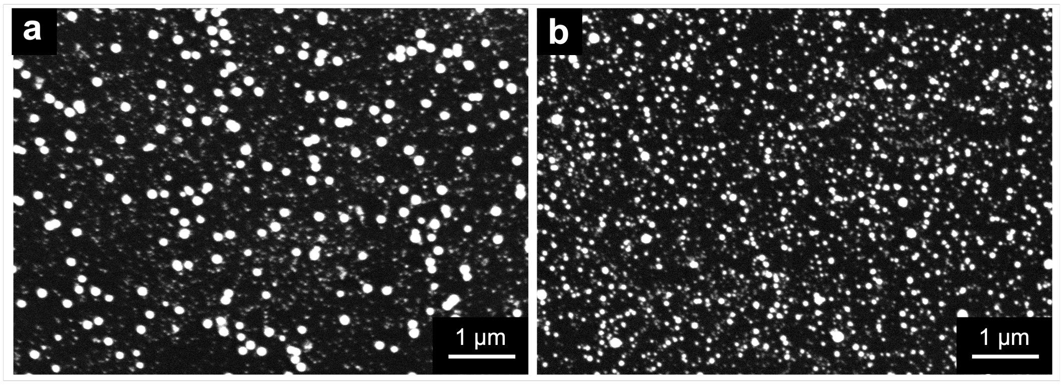

Figure S13. SEM images of sequentially electrodeposited bimetallic nanoparticles. (a) Cu@Pt and (b) Pt@Cu nanoparticles.

S10. Electrodeposition of a Cu-Pt nanoparticle array

A Cu-Pt nanoparticle array comprising 370 depositions is generated using SECCM, as shown in **Figure S14**, highlighting the scalability of this approach. Increasing the pipette size to tens of microns further enhances the number of nucleation sites, significantly boosting nanoparticle yield, similar to the behavior observed in bulk electrodeposition.

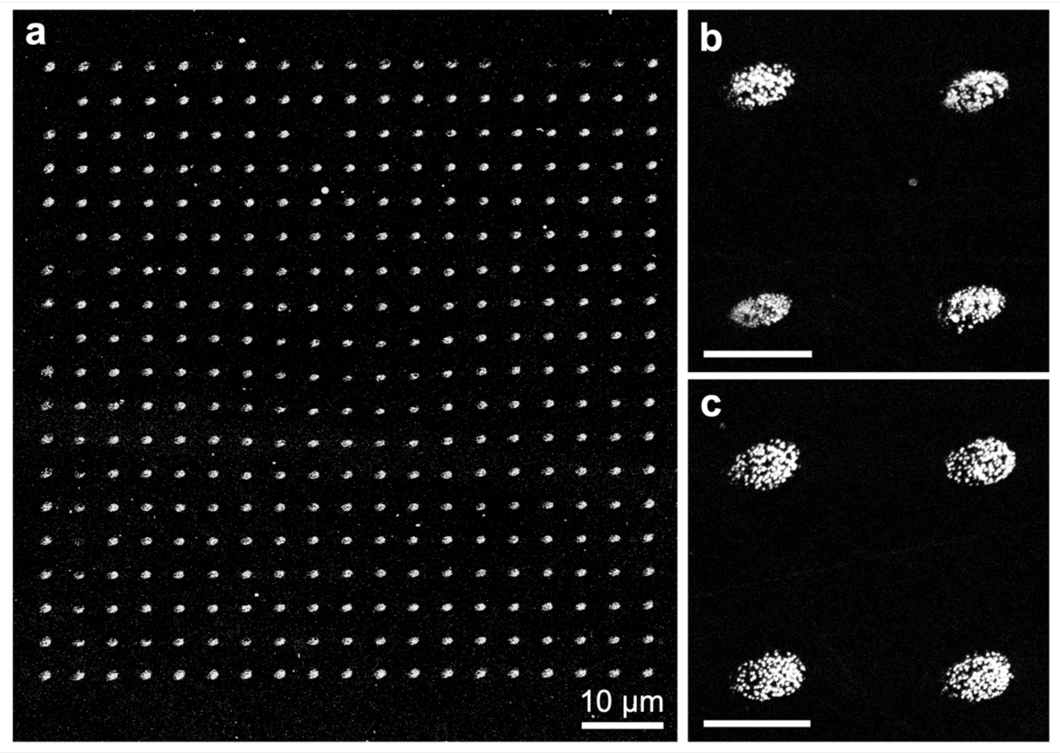

Figure S14. Electrodeposition of a Cu-Pt nanoparticle array on a glassy carbon substrate by SECCM. (a) SEM image showing an array of 19 × 19 depositions using scanning electrochemical cell microscopy (SECCM). (b, c) Magnified SEM images highlighting individual nanoparticle depositions (scale bars: 2 μm). The dual-channel pipette contained 1 mM $Cu(ClO_4)_2$, 1 mM K_2PtCl_4, and 2 mM $HClO_4$ in one channel, while the other channel was filled with 2 mM $HClO_4$. V_{WE} of -1.1 V, V_{bias} of 0.4 V and deposition time of 0.2 s were used for nanoparticle array deposition.

S11. Quantitative comparison of throughput between SECCM and conventional techniques

SECCM configuration has its ability to synthesize an array of nanoparticles with controlled compositions and core-shell hierarchies. Even without deliberate optimization, individual core-shell nanoparticles at a given position can be synthesized in under a second (e.g., 0.2 seconds for core deposition followed by 0.2 seconds for shell deposition, as shown in Figure 1). Including the pipette moving time between substrate locations—up to 6 seconds (but can be easily reduced to 1 s) —each type of nanocatalyst can be produced within approximately 7 seconds. Furthermore, the deposited nanoparticles are readily available for screening, requiring only a replacement of the nanopipette.

In contrast, conventional methods such as wet chemistry or bulk electrodeposition often involve multiple steps to fabricate a single type of core-shell structure. Wet-chemical synthesis might require at least 30 minutes to several hours for each step (including precursor solution preparation, mixing, heating, cooling, and post-processing), resulting in total synthesis times of several hours. For instance, one reported method for Pt-Cu core-shell nanoparticle synthesis involved 2 hours of stirring, followed by vacuum drying at 70°C for 3 hours and subsequent hydrogen treatment of the dried samples for 2 hours at 150–250°C (*Nano Lett.* **2016**, 16, 1, 781–785).). Subsequently, several hours might be needed to load the nanoparticles to the electrode surface before screening can be performed and to remove the ligands. Bulk electrodeposition can also involve multiple sequential steps—each requiring additional time for solution preparation and changing, electrode washing—further extending the overall process. For example, one work was reported for multi-layered Pt/Pd films with switching the precursor solutions of Pt and Pd (*J. Am. Chem. Soc.* **2012**, 134, 26, 10819–10821).

S12. References

(1) Bard, A. J.; Faulkner, L. R.; White, H. S. *Electrochemical Methods: Fundamentals and Applications*, Third edition.; Wiley: Hoboken, NJ, 2022.
